# Supplementary figures and images for: DeepCellState: An autoencoder-based framework for predicting cell type specific transcriptional states induced by drug treatment
Source: PLoS Comput Biol. 2021 Oct 5;17(10):e1009465. doi: 10.1371/journal.pcbi.1009465 (PMC8519465; doi:10.1371/journal.pcbi.1009465)

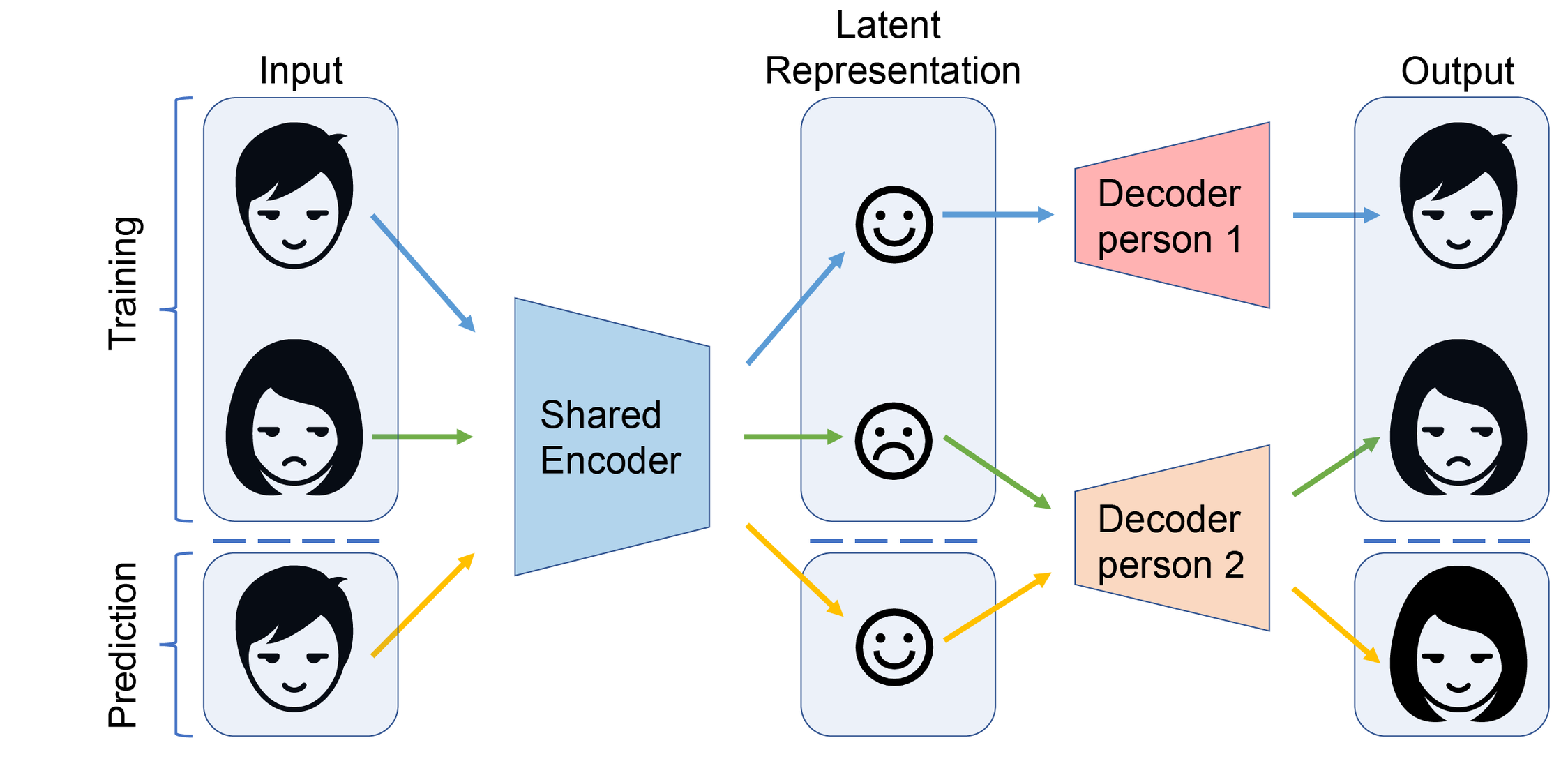

Supplement: S1 Fig — Similar facial expressions are encoded in a similar way in the latent space, while person specific facial details are reconstructed on the decoder side. (TIF) [file pcbi.1009465.s001.tif]

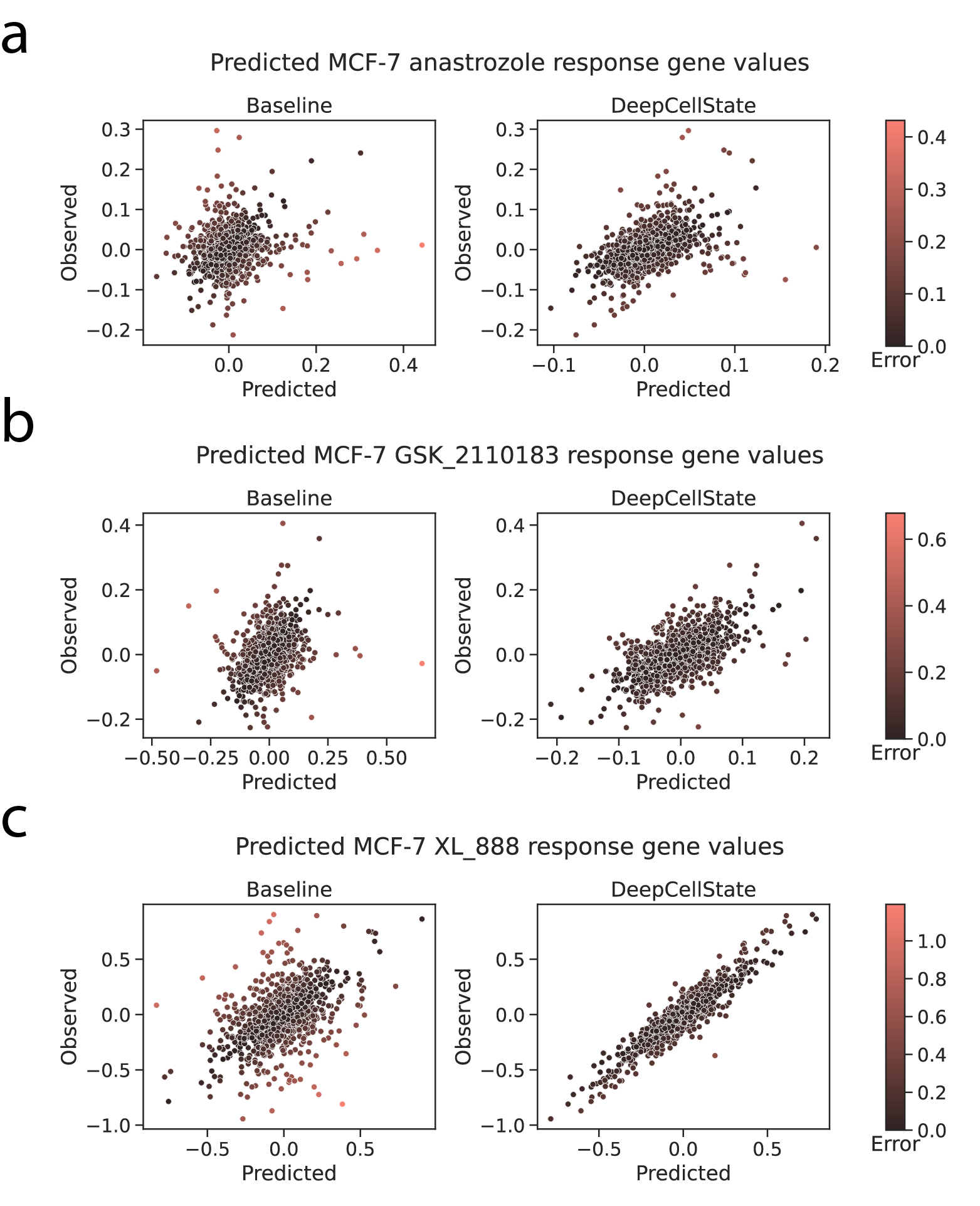

Supplement: S2 Fig — (A): MCF-7 anastrozole response predicted with PCC 0.47. (B): MCF-7 GSK 2110183 response predicted with 0.61 PCC. (C): MCF-7 XL 888 response predicted with PCC 0.93. (TIF) [file pcbi.1009465.s002.tif]

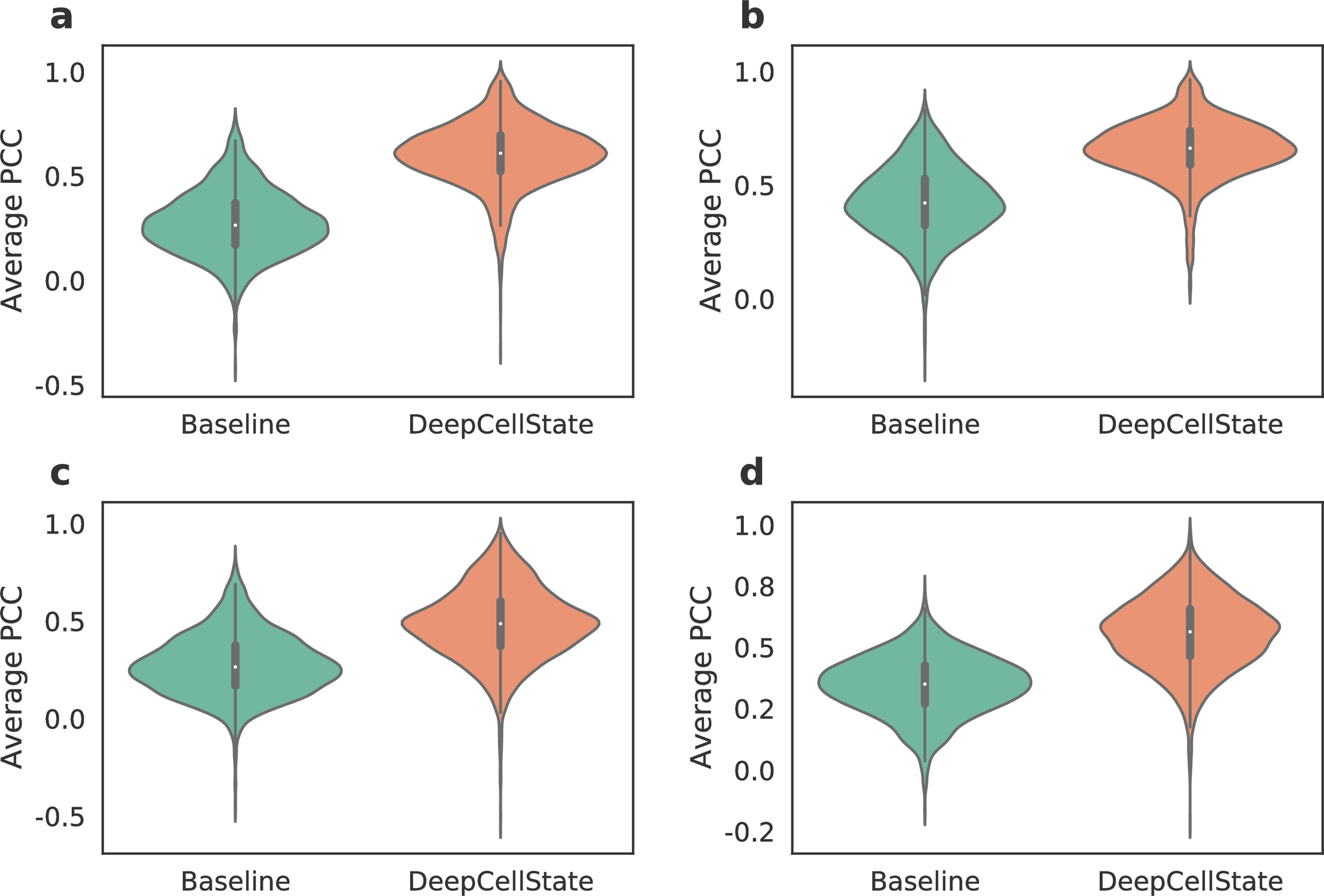

Supplement: S3 Fig — (A): Results obtained by holding out entire drug families for the testing. (B): Performance of DeepCellSate by inclusion of profiles from other cell types in the training set. (C): Performance evaluation using completely unseen cell type as input. (D): Results for shRNA for LoF experiments profiles prediction. (TIF) [file pcbi.1009465.s003.tif]

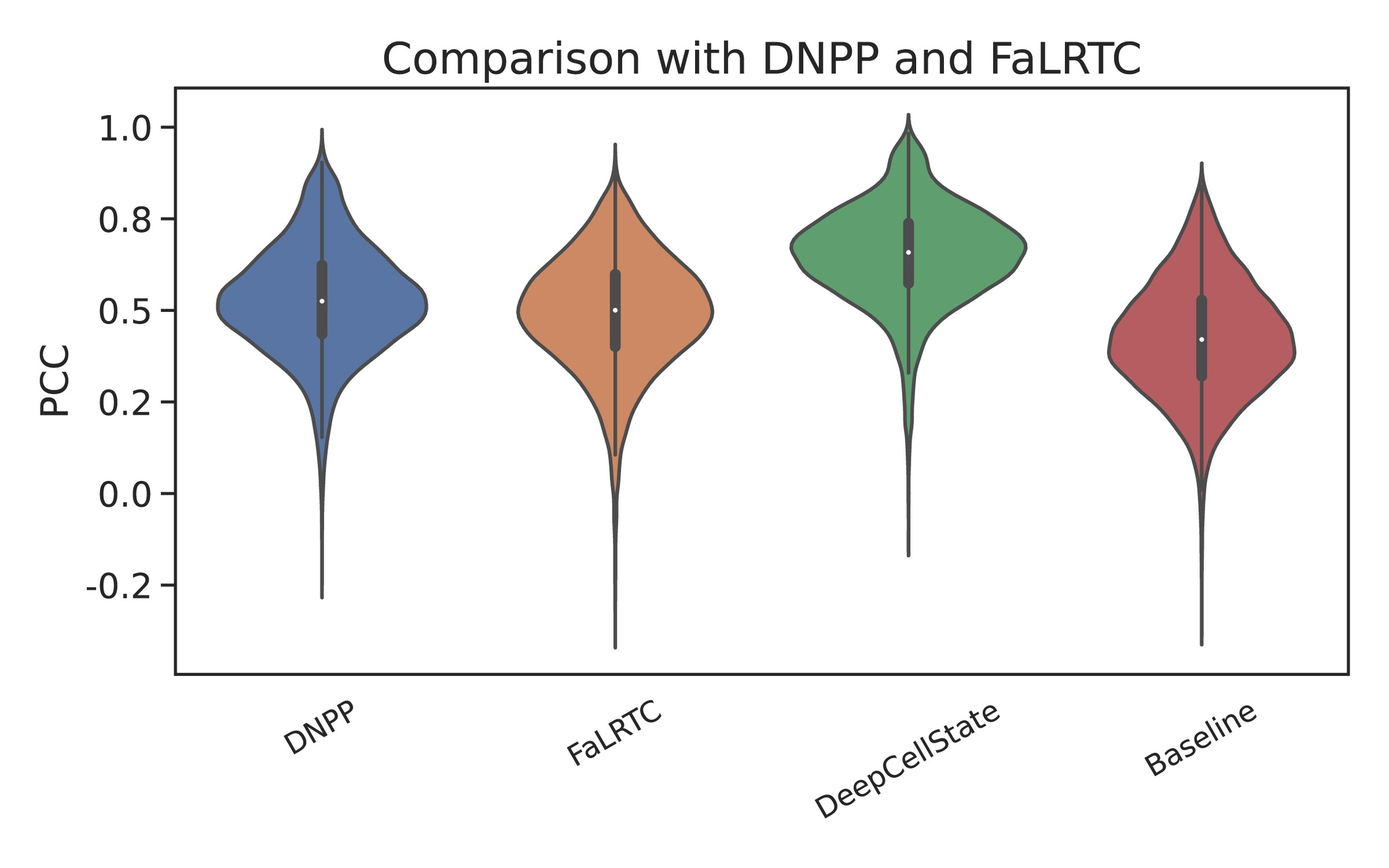

Supplement: S4 Fig — (TIF) [file pcbi.1009465.s004.tif]

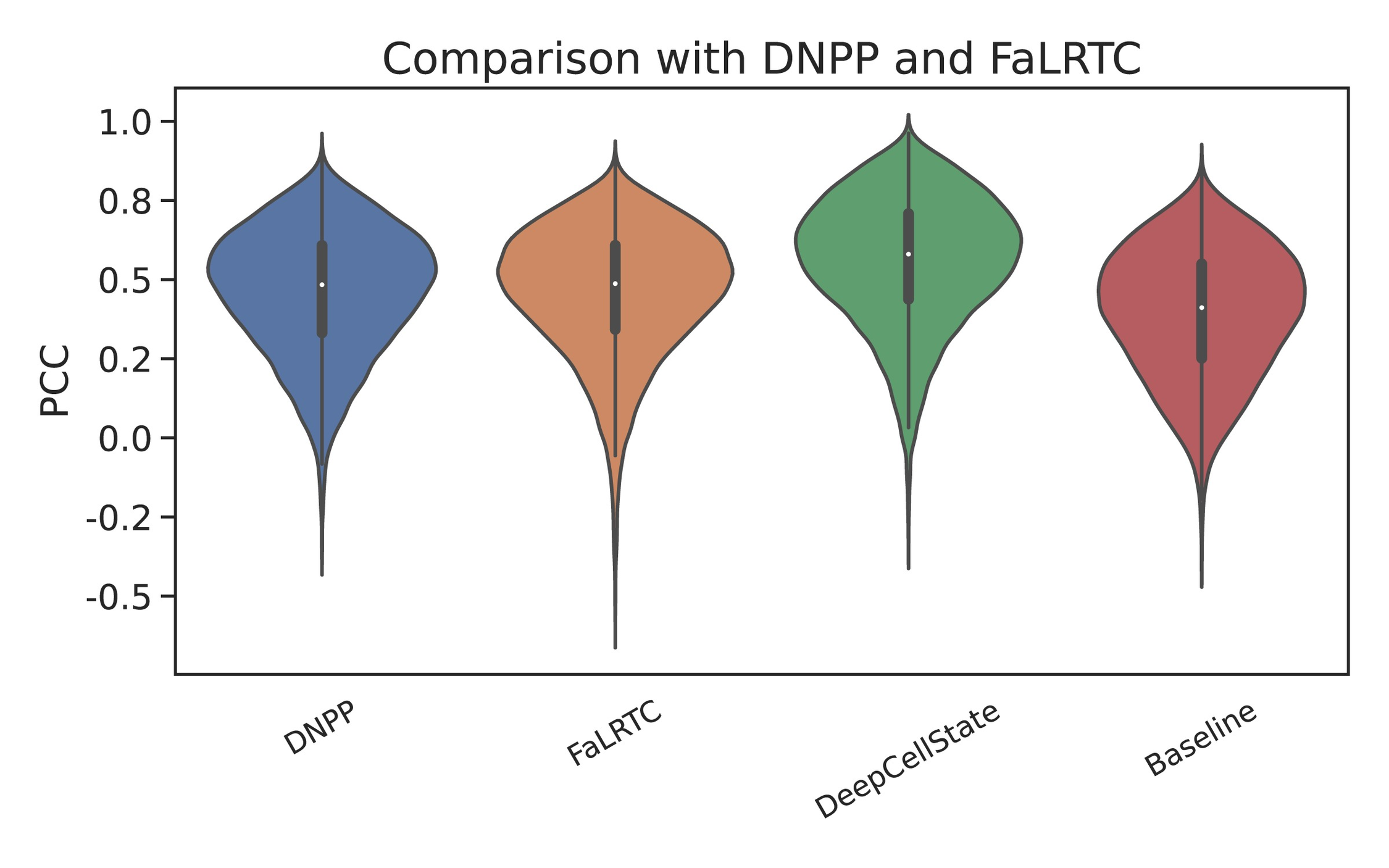

Supplement: S5 Fig — (TIF) [file pcbi.1009465.s005.tif]
